# Sustainable Entrepreneurship Emergence as Practice: A Multi-Level Pathway Model

**DOI:** 10.12688/f1000research.171837.1

**Published:** 2025-11-17

**Authors:** Glenn Pardede, Rosdiana Sijabat, Rizaldi Parani, Jacob Donald Tan, Johana Ataupah, Agung Heru Yatmo, Sekar Dianwidi Bisowarno

**Affiliations:** 1 Faculty of Economics and Business, Pelita Harapan University, Tangerang, Banten, Indonesia; 2 Department of Business Administration, Atma Jaya Catholic University of Indonesia, South Jakarta, Special Capital Region of Jakarta, Indonesia; 3 Faculty of Social and Political Science, Pelita Harapan University, Tangerang, Banten, Indonesia; 4 Independent Researcher, Purwakarta, Indonesia

**Keywords:** Sustainable entrepreneurship, hydroponics, urban agriculture, Jabodetabek, Global South

## Abstract

**Background:**

Sustainable Entrepreneurship (SE) has grown as an emerging theory from the conceptual roots of entrepreneurship and sustainability. While its relevance is increasingly acknowledged in both scholarship and practice, SE remains theoretically young, particularly in how it accounts for the real-world emergence of SE ventures across diverse and underrepresented contexts. This study addresses that gap through a qualitative multiple-case analysis of hydroponic ventures in the urban agriculture sector of Jakarta Metropolitan Area (Jabodetabek), Indonesia.

**Methods:**

Guided by a constructivist paradigm, we collected data through in-depth interviews, field observations, and document analysis. Thematic analysis was conducted across four analytical levels: individual, process, firm, and contextual environment.

**Results:**

This study identifies eight key themes that reflect the ‘how’ and ‘why’ SE emerges in the lived experiences of the hydroponic entrepreneurs. Engagement with existing theories leads to three conceptual propositions that contribute to a grounded understanding of SE emergence. Our findings reveal an alternative path to SE that departs from the intention-led models dominant in the extant literature. Rather than beginning with strategic sustainability goals for the broader society, SE in these cases is triggered by personally meaningful concerns and evolves through insurgent, organic growth, and effectuation logic.

**Conclusion:**

Based on these insights, we develop a multi-level pathway model that illustrates two SE emergence pathways, whether intentional or reflexive. We offer an alternative to prevailing models that assume predefined sustainable entrepreneurial intention.

## Introduction

The concept of Sustainable Entrepreneurship (SE) is in a developing phase and continues to evolve, attracting growing research attention. Although publications have surged since 2009 ( Terán-Yépez et al., 2020; Rosário et al., 2022), the field remains fragmented, with most studies originating in the Global North. This imbalance leaves a gap in understanding how SE unfolds in underexplored contexts, such as developing countries ( Terán-Yépez et al., 2020; Contreras & Dornberger, 2023). As its theoretical concepts are still nascent, SE offers an ideal context between sustainability and entrepreneurship in such settings. This study addresses that gap in the urban agriculture (UA) sector, namely hydroponics, in Jabodetabek, Indonesia, a representative Global South metropolis.

Hydroponics is increasingly recognized as a sustainable solution to urban food challenges ( Khan et al., 2024). These systems meet rising demand for fresh, local produce while reducing environmental impact through resource-efficient technologies ( Barbosa et al., 2015; Pomoni et al., 2023). In Indonesia, hydroponic ventures typically operate as small-to-medium-sized enterprises within informal institutional environments, and have demonstrated strong economic performance, with reported internal rate of returns exceeding 30% and payback periods of less than a year ( Aini et al., 2024; Radhy et al., 2024; Setiyawan et al., 2025). Their capacity to integrate economic viability with social and environmental benefits positions Jabodetabek hydroponics as a compelling empirical site for examining SE practice under uncertain formal structures. Existing SE models may not fully capture the dynamics, given their limited global applicability and development within stable Global North contexts.

To delineate the SE concept, this study utilizes a multi-level approach comprising individual, process, firm, and contextual environment levels ( Spence et al., 2011; Zahra et al., 2014) to ask, ‘How is SE experienced and managed by small-to-medium-sized hydroponic entrepreneurs in Jabodetabek?’. Guided by a constructivist paradigm, we adopt a qualitative case study design to explore the real-world dynamics of UA inductively ( Yin, 2018; Merriam & Grenier, 2019). The existing SE models (e.g., Belz & Binder, 2017; Maaßen et al., 2023) served as sensitizing concepts to guide theoretical interpretation. Data were collected through semi-structured interviews, document analysis, and field observations.

## Literature review

### Definitions of sustainable entrepreneurship

SE integrates the value-creating logic of entrepreneurship with the ecological and social imperatives of sustainability. Drawing on entrepreneurship’s focus on opportunity recognition and innovation ( Shane & Venkataraman, 2000) and sustainability’s emphasis on systems thinking and intergenerational equity ( Purvis et al., 2019), SE reconceptualizes entrepreneurial actions as a means to preserve nature and communities through the realization of a successful business ( Dean & McMullen, 2007; Cohen & Winn, 2007; Hockerts & Wüstenhagen, 2010; Schaltegger & Wagner, 2011; Shepherd & Patzelt, 2011). Unlike social- or eco-preneurship, which prioritize one non-economic goal, SE explicitly integrates profit as both means and ends to enable ecological and social impact ( Muñoz & Dimov, 2015; Bonfanti et al., 2024). In this study, SE is defined as a process through which individuals or organizations recognize, develop, and exploit opportunities that simultaneously aim to generate economic returns, address social needs, and reduce ecological harm.

### Key theoretical frameworks in sustainable entrepreneurship

**Opportunity recognition in SE**

Opportunity recognition is a foundational concept in entrepreneurship, traditionally defined as the discovery or creation of new goods and services that yield economic value ( Shane & Venkataraman, 2000). In SE, this concept expands to include opportunities that also generate social and environmental value ( Dean & McMullen, 2007; Shepherd & Patzelt, 2011). Among the most influential models, Patzelt & Shepherd (2011) define SE as “the discovery, creation, and exploitation of opportunities to create future goods and services that sustain the natural and/or communal environment and provide development gain for others”.

Two key factors shape sustainable opportunity recognition: motivation and knowledge. In SE, motivation goes beyond financial return, incorporating altruistic concerns that increase sensitivity to environmental and social issues ( Segal et al., 2005; Shepherd & Patzelt, 2011). When entrepreneurs perceive threats to valued natural or communal environments, such concerns can drive opportunity recognition. Alongside motivation, prior knowledge plays a central role in enhancing the ability to identify sustainability-related opportunities ( Shane, 2000; Shepherd & Patzelt, 2011).

SE reframes opportunity recognition as a purpose-driven act: rather than responding to market demand, entrepreneurs address unmet societal or ecological needs. This perspective aligns with Belz & Binder (2017), who argue that SE often begins with problem awareness rather than opportunity identification. Despite the strength of such conceptual models (e.g., Cohen & Winn, 2007; Shepherd & Patzelt, 2011), empirical research on SE opportunity recognition remains limited, particularly in the Global South. In these settings, opportunity recognition is shaped by survival needs, cultural expectations, or informal knowledge networks, adding layers of complexity absent in high-income environments.

**SE process**

While individual-level models, such as Shepherd & Patzelt’s (2011), offer insight into how sustainable opportunities are recognized, they do not fully capture how such opportunities evolve into ventures. To address this gap, scholars have developed process-based models of SE. Muñoz & Dimov (2015) conceptualized two distinct pathways through which SE can unfold: the conformist and the insurgent. The conformist path represents the more conventional route, where entrepreneurs start with a clear sustainability-oriented intention and align it with established sustainability frameworks. In contrast, the insurgent path reflects a more emergent and personal journey, where sustainability gradually emerges through experience, reflection, and problem engagement. These dual pathways illustrate that SE does not follow a uniform trajectory but varies in how and when sustainability is integrated.

Complementing this perspective, Belz & Binder (2017) propose the Convergent Model, developed through qualitative case studies of early-stage SE ventures in Germany and Finland. Integrating entrepreneurship theory with sustainability thinking, the model outlines six phases: (1) Recognizing a social or ecological problem, (2) Recognizing a corresponding opportunity, (3) Developing a double bottom line (DBL) solution, (4) Developing a triple bottom line (TBL) solution, (5) Funding and forming the sustainable enterprise, and (6) Creating or entering a sustainable market.

This structure is significant for three reasons. First, it distinguishes between problem and opportunity recognition, acknowledging that many entrepreneurs begin with awareness of a pressing issue rather than a business idea, suggesting that SE is often mission-driven rather than market-led. Second, the model captures stepwise integration of sustainability goals, as entrepreneurs often begin pursuing a DBL before fully integrating the TBL, reflecting the realistic constraints of early-stage ventures. Third, it highlights the role of broader institutional and market dynamics, positioning SE not just as an individual act but as embedded in wider ecosystems.

Other notable models include Matzembacher et al. (2020) and Maaßen et al. (2023). In our study, these models serve as sensitizing frameworks to interpret how hydroponic entrepreneurs in Jabodetabek develop their ventures around sustainability concerns. Rather than applying it prescriptively, they guide the analysis flexibly, allowing adaptation to the Global South context of UA while retaining its conceptual strengths.

**SE in global context: North–South divergence**

This framing reflects long-standing global inequalities that influence how knowledge, innovation, and development are conceptualized and practiced. These divides are evident in SE, where dominant theories and empirical models have emerged primarily from Global North contexts. Although SE is often presented as a universal framework, its assumptions largely reflect high-income contexts where it is opportunity-driven and supported by infrastructure, finance, and regulation that enable alignment with global standards such as the SDGs ( Schaltegger & Wagner, 2011; Bocken et al., 2014).

In contrast, much entrepreneurial activity in the Global South is necessity-driven, shaped by poverty, informality, and institutional voids ( Mair & Marti, 2009). Sustainability is often embedded in everyday strategies for survival and resilience rather than formal objectives. Entrepreneurs rely more on informal networks, local knowledge, and adaptive practices than on global sustainability discourse.

This imbalance is also evident in academic production. Over 60% of SE publications originate from the Global North, focusing on environmental metrics, technology, and policy tools, while underrepresenting the lived realities of Southern entrepreneurs ( Contreras & Dornberger, 2023). Research grounded in the Global South remains underfunded and under-cited, reinforcing epistemic asymmetries in how SE is theorized ( Terán-Yépez et al., 2020). The result is twofold: context-specific Northern models are universalized, and alternative sustainability practices, emerging from constraint, improvisation, or collective adaptation, are obscured. Addressing this divide requires broader empirical representation and critical reflection on SE’s normative assumptions. Table 1 outlines these key differences across North–South contexts ( Mair & Marti, 2009; Terán-Yépez et al., 2020; Contreras & Dornberger, 2023).

**Table 1.** Comparative characteristics of SE in the Global North and Global South.

Aspect	Global North	Global South
**Focus**	Sustainability-driven innovation	Survival-driven entrepreneurship
**Infrastructure**	Advanced, with strong institutional support	Limited, with weak policy frameworks
**Themes**	Circular economy, green technologies	Social impact, poverty alleviation, and informal markets
**Academic output**	Dominates with significant influence	Underrepresented, often context-specific
**Collaboration**	Strong intra-regional networks	Weak intra-regional and unequal North-South collaborations

Source: Curated from various sources.

### Urban agriculture and hydroponics

Urban agriculture (UA) has emerged as a critical response to challenges of urbanization, food insecurity, and environmental degradation. As cities expand and food systems globalize, UA is seen to address economic, social, and environmental concerns through locally rooted solutions ( Orsini et al., 2013; Lal, 2020). Broadly defined, UA includes the cultivation, processing, and distribution of food and non-food products in and around urban areas, often using urban resources for production ( Food and Agriculture Organization, 2007; de Zeeuw et al., 2011; Lal, 2020).

UA has gained scholarly attention for its role in enhancing food security, generating income, and promoting ecological resilience, especially in the Global South, where formal food systems may be less accessible ( Dubbeling et al., 2010; Khan et al., 2024). It also contributes to global agendas, including the SDGs, addressing hunger (SDG 2), sustainable cities (SDG 11), and climate action (SDG 13). Increasingly, UA is recognized not only as a food strategy but also as a platform for SE.

Hydroponics, a method of growing plants using water-based nutrient solutions, is one of the most prominent forms of UA ( Resh, 2012). It is well-suited to urban environments with limited or degraded land, offering high productivity and efficient resource use ( Asao, 2012; Barbosa et al., 2015; Magwaza et al., 2020). Controlled conditions also support faster growth, higher yields, and year-round production ( Barbosa et al., 2015; Naresh et al., 2024). These features make hydroponics a relevant model for sustainable UA in both the Global North and South. In dense cities, such as Jabodetabek, hydroponics provides a promising alternative to soil-based farming ( Martinez & Masron, 2020; Naresh et al., 2024).

## Methods

### Research paradigm

We adopted a constructivist paradigm to guide this study, as it aligns with our aim to understand the subjective realities of hydroponic entrepreneurs in a developing country. Constructivism views reality as co-created through human experience and interaction rather than as an objective truth ( Schwandt, 1994). Consistent with this paradigm, a qualitative approach was employed to explore how hydroponic entrepreneurs in Jabodetabek perceive and engage with the complexities of SE within UA. Qualitative inquiry, grounded in inductive reasoning, enables us to uncover meaning and process as they emerge from participants’ experiences ( Merriam & Grenier, 2019). To further guide our exploration, we used sensitizing concepts such as the SE Process Model ( Belz & Binder, 2017; Maaßen et al., 2023) as flexible frameworks for interpreting SE within this context.

### Research design

To address the research questions, we employed a multiple-case holistic design ( Yin, 2018) to examine several hydroponic ventures across Jabodetabek. All informants were (co-)founders who directly experienced the development and management of their enterprises. This design enabled cross-case analysis to identify patterns and variations in how these entrepreneurs navigated the challenges and opportunities of SE in urban hydroponic farming.

### Case study procedure

To ensure that the cases reflected commercially viable and contextually relevant hydroponic enterprises, three purposive criteria were applied: an operational scale of between 10,000 and 750,000 planting holes, a minimum operational duration of three years, and a location within Jakarta, Bogor, Depok, Tangerang, or Bekasi. These criteria targeted small-to-medium-sized enterprises actively engaged in SE practices, excluding hobbyist or large corporate ventures whose objectives differed from the study’s focus on opportunity recognition ( Belz & Binder, 2017; World Bank, 2019a, 2019b).

A pilot study conducted in April 2022 with 25 stakeholders—including hydroponic practitioners, NGOs, academics, and government representatives—helped refine these selection criteria, regional focus, and data collection instruments ( Pardede, 2022). Four hydroponic businesses were subsequently selected, with data collected between April and August 2024 through semi-structured interviews with their (co-)founders, each lasting 1-2 hours and complemented by on-site observations and document reviews. Data saturation was reached after the fourth case.

### Data analysis

We adopted an inductive and iterative approach to analyze the data, guided by Crabtree & Miller’s (1992, as cited in Wahyuni, 2024) editing analysis style, complemented by Charmaz’s (2014) constructivist grounded theory principles and Yin’s (2018) cross-case synthesis and explanation-building techniques. This combination allowed us to maintain analytical flexibility while ensuring rigor in developing themes across cases. Our goal was to build theoretical rather than statistical generalizations ( De Vaus, 2001), deepening the understanding of how SE emerges and evolves within urban hydroponic farming in Jabodetabek.

All interviews were transcribed in Indonesian to preserve cultural nuances, and we used NVivo software to support systematic coding while remaining closely engaged with the data. The initial phase involved word-by-word and line-by-line coding to identify emerging concepts and patterns. We then refined these codes into broader conceptual categories, linking them with relevant theoretical frameworks to develop analytic propositions. This iterative process ensured that our interpretation remained contextually grounded while contributing to the broader SE discourse.

We conducted cross-case analysis using pattern matching to identify both commonalities and variations among hydroponic enterprises, which helped us understand how entrepreneurs navigate different SE trajectories. Verification involved constant comparison and triangulation across interviews, observations, and documents to ensure consistency and validity.

## Results

Four hydroponic enterprises in Jabodetabek are selected as cases, each closely tied to its (co-)founder (i.e., A, D, T, and J), reflecting the personal and small-scale nature of SE in the region. Key entrepreneurial dimensions are summarized in Table 2 and Table 3. Each case was thematically analyzed, and continued with cross-case analysis, resulting in eight related themes ( Table 4) as research findings.

**Table 2.** Profile of the (Co-)founders.

Name	Age range	Previous occupation (Background)	Income Contribution of hydroponics	Other occupation
A	40-50	Sales in the furniture industry	Main income source (has other side income)	Hydroponic trainer/consultant and still active in a sales job
D	45-55	Quality control in the automotive industry	Sole income	None (full-time in hydroponics)
T	50-60	Shipbuilder and captain	Main income source (has other side income)	Occasionally helps others in logistics
J	25-35	Mechanical worker in the automotive industry	Sole income	None (full-time in hydroponics)

Source: Own elaboration from research data.

**Table 3.** Profile of the hydroponic ventures.

Name	Location	Started experimenting with hydroponics	Started selling produce	Scale (Planting holes)	Initial capital source	Business form	Market scope	Main types of vegetables grown	Estimated monthly revenue (IDR)
A	Tajur Halang, Bogor	2017	2019	30,000	Personal savings, and still maintaining a regular job	Private, small-to-medium-sized enterprise, family-run operation	Local communities, Pita Pink, resellers (B2B partners)	Lettuce, pakcoy	30-45 million
D	Cijeruk, Bogor	2014	2020	150,000	Personal savings (from pension), and later, expanded through a partnership	Private, small-to-medium-sized enterprise, run with a business partner	SP, local communities (occasionally)	Lettuce, pakcoy, spinach	150-225 million
T	Bojongsari, Depok	2016	2017	14,000	Personal savings	Private, small-to-medium-sized enterprise, family-run operation	Reseller (B2B partners)	Spinach, kangkong	15-20 million
J	Babelan, Bekasi	2020	2021	25,000	Personal savings and parent support; later secured a bank loan to expand the operation	Private, small-to-medium-sized enterprise, solo-operated	Local communities, resellers, B2B partners)	Pakcoy, spinach, kangkong	25-35 million

Source: Own elaboration from research data.

**Table 4.** Results of thematic analysis.

Themes	Sub themes	Cross-case summary			
		A	D	T	J
1. Starting from personal concerns	Initial trigger	Wife’s health: limited access to pesticide-free vegetables	Discomfort in the workplace	Wife’s hobby and unused backyard	Curiosity after watching a viral YouTube video
	Orientation	Own family	Own self	Own family	Own self
	Initial intent	Provide safe vegetables for the family	Find peace by being close to nature and having a sense of control	Utilize the house yard meaningfully for wife’s gardening interest	Compelled to prove the replicability and simplicity of the system
2. Recognizing opportunity in everyday setting	Initial framing of hydroponic	Hydroponics for healthy vegetable production for the family	Hydroponics as a self-managed activity with direct contact to nature	Hydroponics as a meaningful land utilization	Hydroponic replication as a proof-in-action response to viral tutorials
	Triggering feedback	Interest from community outreach	Neighbor diner started buying produce	Positive reception from friends/neighbors	Neighbors praised the quality and asked to buy
	Form of validation	Shared health concern	Unprompted local purchase	Encouraging responses	Quality affirmed informally
	Reframed as an opportunity	From family care to economic potential	From hobby to economic potential	From the household use economic potential	From personal trial to economic potential
3. Building a viable practice through experimentation	Initial learning source	Self-learning, peer observation	Self-learning, online source, peer observation	Self-learning, peer observation	YouTube, Self-learning
	Technical trial	Backyard trials with family, trial at *Pesantren* with students	Two growing racks at home	Small garden at home	Three growing racks at home
	Market engagement trial	Interest from the Pita Pink community, friends, resellers, and vegetable stores	Neighboring *warung*, surrounding households, and the wet market	Friends and community guests, wet market	Neighbors, Resellers, Modern Markets (experiment with renting stalls)
4. Sustainability emerging in practice	Environmental values	Pesticide control, high land efficiency, soil independence, low waste, low emission from local production	Controlled system + gravity-fed irrigation system, low organic waste, connection to nature	Land space utilization, waste reduction, connection to nature, greenery	Flood resilience, reclaimed land, automated irrigation (water saving), and minimal pesticide use
	Social values	Funded *Pesantren* students, hosted vocational interns, shared vegetables and waste	Shared knowledge with youth, employed low-skilled labor	Shared space and vegetable, modern farming experience, youth engagement	Opened site to interns, shared surplus, jobs for unskilled workers, youth engagement, shared space for gatherings
	Economic values	For customer benefits: Affordable, nutritious, high-quality and tasty produce for health-conscious consumers. For own self: Supplementary income, vegetables for self-consumption	For customer benefits: High-quality, tasty, locally produced vegetables. For own self: Career switch	For customer benefits: Accessible, high-quality & nutritious vegetables. For own self: Supplementary income (toward main income in future)	For customer benefits: Fresh, high-quality vegetables with better taste and a longer shelf life, offered at a reasonable price, grown in the neighborhood. For own self: Career switch
5. Building trust while meeting needs	Trust-building practice	Customized produce, spousal division of labor, and informal selling	Attention to the quality of produce, including packaging and supply	Limited client base, prioritized quality	Price stability, local availability
	Balance of needs	Community nutrition and contribution to *Pesantren* vs. family livelihoods	Customer satisfaction vs. reliable income and personal peace	Avoided overextension and ensured crop quality vs. time with family	Neighbor and community support (local affordability) vs. income independence
6. Managing costs with ingenuity	Examples of adaptation methods	Dual cropping, cooling with stones and a fan, local media and seeds, local & low-skilled worker	Gravity-fed irrigation, semi-open screen house, local & low-skilled worker	Composting, reused waste, DIY nutrients, local & low-skilled worker	Automated irrigation, reused materials, simple packaging, local & low-skilled workers
	Purpose/logic	Affordability, replicability	Energy savings, simplicity	Cost-saving, reuse	Cost, time saving, minimalism
	Outcomes	Efficient system, lower input cost, replicable setup, teaching materials for training	Reduced costs, easy maintenance	Lower expenses, harvests from other crops	Operational ease, customer recognition retained
7. Managing with simplicity	Examples of management practices	Shared tasks with spouse, adjusted planting cycles, simple workforce & strategy	Manual records, self-trained workers	Limited product range, uses a distributor	Standardized packaging (no label) and pricing (flat)
	Simplicity logic	Align with capacity and health goals. Aim for consistent and sustained production	Avoid complexity, easier oversight (reduce stress) and smooth operations	Prevent overload, protect family time, stable quality, consistent supply	Minimize decision making, time efficiency, consistent daily routine, system clarity
8. Expanding gradually and organically	Market expansion	Community networks (local and online market), B2B	Partnership with B2B distributors	Partnership with B2B distributors	Community networks, B2B distributors & modern markets
	Operational	Expanded plots and crop types	Expanded plots and crop types	Crop layout adjustments	Expanded sites and crop types
	Service expansion	Providing formal and informal training and consultation, hosting vocational interns	Envisioning a youth training center	Hosting vocational interns	Opening the garden for community programs and vocational interns

Source: Own elaboration from thematic analysis.

## Discussion

Building on the thematic findings ( Table 4), we proceed by interpreting them through relevant theoretical lenses and formulating conceptual propositions. The analysis is organized across four levels (individual, process, firm, and contextual environment). Each concludes with a proposition intended to contribute to a more grounded understanding of SE in the context of hydroponic entrepreneurs in Jabodetabek.

### Individual-level insights (Themes 1, 2, and 5)

At the individual level, this study offers a key insight: SE need not begin with an explicit sustainability intention. It can emerge organically from inward-facing, personally meaningful problem-solving (Themes 1 and 2). This challenges the dominant assumption that SE is inherently driven by outward-oriented missions or SDG agendas; a desire to, in Linnanen’s (2002) words, ‘change the world’.

Shepherd & Patzelt (2011) illustrate the outward-facing moral impetus through cases such as Muhammad Yunus’s Grameen Bank, which was founded to remedy exploitative micro-lending among Bangladesh’s poorest. Likewise, Belz & Binder (2017) document SE ventures tackling systemic issues: energy poverty through decentralized renewables, textile waste to protect ecosystems, unfair coffee trade to secure farmer livelihoods, and digital monopolies to democratize e-commerce. In all of these, the founders directed their entrepreneurial energy toward broader social or ecological well-being rather than seeking personal gain. In sharp contrast, the hydroponic entrepreneurs in this study initiated their ventures in response to problems affecting their own lives, not out of duty to external causes. This divergence challenges the tendency to equate SE exclusively with outward-oriented, mission-driven action.

In our study, the traits commonly associated with SE entrepreneurs, i.e., cognitive alertness and prior knowledge, were evident but operated differently. Alertness was directed inward, toward personally relevant disruptions, and actions were oriented toward self-serving goals ( *change one’s own world*, instead of *change the world*). Opportunity recognition unfolded as an extension of daily routines, without deliberate pursuit. None of the informants began with entrepreneurial intentions, nor did they consciously frame their actions as sustainability contributions. Prior knowledge informed what felt feasible and familiar but did not reflect awareness of sustainability issues. Trust, rather than serving as a scaling mechanism, functioned as a relational practice of care and reciprocity, fostering resilience but potentially constraining growth.

Proposition 1: A personal problem-solving orientation grounded in personally meaningful concerns, without entrepreneurial intention or predefined sustainability goals, can lead to the emergence of SE.

These insights contribute to SE theory in three ways. First, they demonstrate that SE can begin without an initial sustainability logic, emerging instead through personal meaning-making and everyday action. Second, they reframe opportunity recognition as a process of situated responsiveness, where problems are acted upon rather than ideated in advance. Third, they extend the insurgent path ( Muñoz & Dimov, 2015) by showing that such ventures can grow not from ideological resistance, but from relational, context-bound engagement. This proposition opens space to explore how inward-facing entrepreneurial processes, which respond meaningfully to one’s own world, can unfold into SE trajectories, and how such personal orientations interact with broader dynamics across the SE journey.

### Process-Level Insights (Themes 2, 3, 4, 5, 6, and 8)

Van de Ven (1992) defines a process as a ‘logic of progression’. In entrepreneurship, this is extended to explain how ventures evolve. This study interprets the SE process across two dimensions: the what, referring to recurring qualities of SE practice; and the how, referring to the mechanisms and trajectories through which ventures develop.

**The what: Recurring qualities of SE practice**

The recurring qualities of SE practice (the *what*) observed in this study reflect five key characteristics. First, the hydroponic informants did not begin with a clear intention to become sustainable entrepreneurs. Instead, they acted on unmet needs which come from everyday concerns such as food safety, family health, or land use; and only through continued engagement, did a sense of purpose gradually take shape. This may be termed *reflexive* intention, where entrepreneurial purpose is not predefined but develops through iterative engagement, compared to the commonly regarded as ‘intentionally’ in Belz & Binder (2017). Over time, these informants transitioned from addressing personal concerns to recognizing opportunities through experimentation and refinement of intent.

Second, practice unfolded through continuous experimentation and a gradual shift toward using the results for business purposes. This can be described as *practice-led opportunity formation.* It did not emerge from prior knowledge or analytic discovery, as theorized by Shane (2000), but through embodied, lived engagement with the entrepreneurs’ environments. This aligns with Muñoz & Dimov’s (2015) argument that opportunities in SE are not always ‘found’ but emerge as extensions of ongoing practices and commitments. In this case, doing preceded framing. Hydroponics became a solution because it worked in the informants’ everyday realities, not because it was selected from a predefined set of options.

A third quality is *contextual embeddedness* in shaping process trajectories. As emphasized by Welter (2011) and Zahra et al. (2014), context is not merely a backdrop for entrepreneurship; it co-constitutes the process. In this study, the environment shaped what was possible and desirable. Household trials, relational market interactions, and trust-based support directed the pace and trajectory of their contextual development.

Fourth, the process also displayed *non-linear adaptation.* Development unfolded in overlapping, recursive cycles rather than discrete, sequential steps. While Belz & Binder’s (2017) model suggests a clear progression from problem recognition to TBL integration, the hydroponic entrepreneurs often experienced blurred transitions and feedback loops between experimentation, realization, and expansion. This aligns with effectuation theory ( Sarasvathy, 2001; Read et al., 2009), which sees entrepreneurial paths as contingent and constructed through action, and with Garud & Karnøe’s (2003) view of path creation as situated improvisation. Hydroponic ventures were frequently located in or near homes, run by family members, and integrated into domestic routines. This everyday orientation contributed to the ventures’ resilience during shocks, such as the COVID-19 pandemic, and aligns with calls for more context-sensitive, situated understandings of SE ( Shepherd & Patzelt, 2011; Giacomini et al., 2016).

At the same time, these adaptive responses were not random or chaotic; they were anchored in consistent motivations. This suggests that SE can be fluid in form yet stable in purpose. These findings further challenge cognitive perspectives in SE that emphasize pre-existing entrepreneurial intentions ( Kuckertz & Wagner, 2010; Shepherd & Patzelt, 2011), and support Muñoz & Dimov’s (2015) view of SE as an evolving enactment shaped by lived concerns. This pattern of overlapping action and reflection highlights a non-linear, adaptive development built through cumulative, relational steps that become clear only in hindsight.

Finally, the developmental process reflected *layered value creation.* Economic, social, and environmental considerations were not strategically balanced from the start but emerged incrementally. This challenges assumptions that SE must be guided by explicit sustainability frameworks (e.g., Schaltegger & Wagner, 2011) and supports a view of sustainability as enacted through habit and value-driven action ( Muñoz & Cohen, 2017). As entrepreneurs encountered real-world tensions and feedback, new directions were incorporated into their evolving practices. In this sense, sustainability functioned less as a label or intention and more as a cumulative outcome.

**The how: Developmental trajectories over time**

In SE literature, at least two contrasting approaches have emerged to explain how the SE process unfolds. One group of scholars, such as Muñoz & Dimov (2015), describe development as an emergent and context-dependent process, distinguishing between conformist and insurgent pathways. On the other hand, Belz & Binder (2017) offer a more structured perspective through a six-stage model of SE development, emphasizing that the TBL is integrated sequentially in a convergent rather than simultaneous manner.

Reflecting on these perspectives, our study suggests that while analytical models may divide SE into discrete phases, the lived experiences of the informants reveal a much more fluid and overlapping reality. Rather than unfolding in clean steps, their process was dynamic, marked by recursive loops, blurred boundaries, and a continual interplay between learning, doing and adapting. Table 5 illustrates the contrast between the Convergent Model and the informants’ lived realities.

**Table 5.** Sensitizing comparison: The convergent model ( Belz and Binder, 2017) and SE practices among hydroponic informants.

Belz and Binder’s convergent model	Observations in informants’ practices	Remarks
Phase 1: Recognizing a social or ecological problem	Reflected in practice (Theme 1)	Informants began with personally meaningful concerns, which were often social but oriented inward toward personal or family needs. This contrasts with the Convergent Model’s focus on outward, systemic problem identification.
Phase 2: Recognizing a social or ecological opportunity	Reflected in practice (Theme 2)	Recognition of opportunity followed naturally from the personal concerns previously identified. However, the entrepreneurial mindset was not yet clearly present.
Phase 3: Developing a DBL solution	Partially reflected (Themes 3 and 4)	- Informants consistently began with a period of small-scale experimentation, essential to surfacing the economic dimension of their solutions. - Economic motivations reflected both customer-oriented and self-benefits. These goals did not stem from a strategic sustainability framework but arose from practical concerns, with social and environmental impacts emerging as byproducts.
Phase 4: Developing a TBL solution	Partially reflected (Themes 4 and 5)	In the Convergent Model, entrepreneurs deliberately integrate the third dimension, after first addressing the other two, typically economic and either social or environmental. This integration is framed as a strategic and conscious step before market entry. In contrast, the informants began with personally meaningful concerns (mostly social), while the ecological aspect was inherently embedded in the hydroponic system. Although all three dimensions eventually emerged, sustainability was adopted through situational practices.
Phase 5: Funding and forming of a sustainable enterprise	Reflected in practice (Theme 6)	Unlike the Convergent Model, where external funding and formal incorporation occur before market entry, the hydroponic informants transitioned gradually from small-scale trials to structured enterprises. - Funding came primarily from personal savings or family support, with no large external seed capital. - Business formation was a flexible, adaptive process influenced by each informant’s circumstances, risk tolerance, and access to resources.
Phase 6: Creating or entering a sustainable market	Reflected in practice (Themes 7 and 8)	Market participation was multi-layered and adaptive. Access unfolded through relationships and iterative service during the trial phase, not formal planning.

Source: Own elaboration.

In the early stages, the lived experience did not follow a linear path from problem recognition to opportunity development. Instead, informants began with personal concerns and moved forward through practical experimentation (Theme 3). These moments of experimentation were crucial because they disrupted any sense of orderly progression. Since the trials were informal, iterative, and lacked predefined goals, they blurred the line between identifying problems and recognizing opportunities. Effectuation theory ( Sarasvathy, 2001) offers a useful lens here, emphasizing means-driven action, flexibility, and the co-creation of goals through ongoing engagement rather than strategic planning. These insights align with the experience of the informants, whose practical experimentation unfolded without a clearly defined endpoint.

The transition from a personal need to a business opportunity did not happen all at once or through deliberate planning. Instead, it unfolded gradually through everyday actions and informal feedback that made sense in the informants’ lives. They reframed a personally relevant concern into a concrete opportunity, often without fully realizing when that shift occurred. Table 6 offers a cross-case summary of how each informant framed hydroponics as an opportunity.

**Table 6.** How hydroponic informants reframed personal concerns into entrepreneurial opportunities.

Hydroponic informants	Identified personal concerns	Opportunity framed through hydroponics
A	Limited access to pesticide-free vegetables	Used hydroponics to produce healthy, pesticide-free vegetables. He began by experimenting to grow vegetables for his family (particularly his wife) and later expanded to serve a broader community with similar needs for healthy produce.	
D	Workplace discomfort	Established a hydroponic business as a potential new occupation that offered a more natural, peaceful, and self-managed work environment. He experimented to test his ability to grow vegetables. Early harvests were supplied to a neighbor’s diner.	
T	Need for meaningful utilization of the house yard for family	Built a hydroponic installation in the yard of the Sawangan house as a meaningful way to use the land for the family. He experimented after the installation was built to test his ability to grow vegetables. The produce was initially intended for sharing with neighbors.	
J	Boredom and influence from online hydroponic influencers	Replicated a viral hydroponic installation as a personal challenge and productive project. He was motivated by curiosity and a desire to test its feasibility. Produce from early trials was sold to nearby neighbors.	

Source: Own elaboration.

The movement between opportunity exploration and value proposition development among the informants was fluid and iterative rather than sequential. Instead of defining a problem and then crafting a solution, they cycled between sensing needs, experimenting with ideas, and gradually recognizing their potential to create value. Lacking clearly defined target customers, they discovered their markets through trial and error, contrasting with the Convergent Model, which assumes entrepreneurs translate social or ecological goals into economic value for specific customer segments. For these hydroponic entrepreneurs, economic goals emerged only after small-scale experimentation and personal use, which marked their transition toward Phase 3: developing a DBL solution.

Notably, the economic benefits were not limited to customers. They also applied directly to the informants themselves. Initial experimentation focused on the technical aspects of hydroponics, and once the systems succeeded in producing vegetables, the produce was primarily consumed by their own households. As trials continued and yields increased, informants began sharing the surplus with close community members. As the vegetables became known and appreciated, interest grew, and the process naturally led to informal confirmation of demand. At certain points, small experiments in selling began to take place. Interestingly, this transition mirrors the emergence of the other two dimensions (social and environmental), which were also not initially directed outward toward customers, but applied first to the informants themselves.

The integration of social and environmental dimensions in the informants’ business models aligns with the Convergent Model, emerging naturally from their ethical and holistic worldviews rather than from legal or market pressures. The key difference lies in the sequence of this integration. In the model, the third dimension is typically incorporated after entrepreneurs have established solutions that address both economic and either social or environmental goals. Among the hydroponic informants, initial opportunities were predominantly social. While economic and environmental aspects did follow, they did not emerge from strategic intent or in a linear sequence, but through ongoing practice. These dimensions appeared simultaneously, often incidentally, and became intertwined with daily business activities (Theme 4). The informants did not begin with clearly articulated ecological objectives. Instead, environmental considerations surfaced organically as they interacted with their hydroponic systems. Thus, although hydroponics inherently offers environmental advantages compared to conventional farming, each entrepreneur experienced and valued these benefits differently, shaped by their own circumstances and business evolution.

Similarly, social benefits accumulated over time, adding to the initial motivations that had first led them to explore hydroponics. While the primary social goals were addressed early on, informants gradually discovered additional social contributions from their hydroponic practices. These included engaging youth and students in agricultural learning, promoting cleaner and more accessible farming methods, and fostering a sense of community through shared spaces, donations, and sustainable waste reuse. The social, economic, and environmental dimensions continued to emerge throughout the entrepreneurs’ journeys, with no clear distinction between planning and execution or between experimentation and established business activity.

This overlapping dynamic extended into the growth phase of the business. All three dimensions evolved simultaneously, with no clear boundaries between stages, including market entry. In the Convergent Model, Phase 6 marks a distinct entry point, where entrepreneurs position their sustainable businesses, secure customers, and expand their market presence. This entry is often strategic and deliberate. In contrast, the hydroponic informants had already experimented with sales approaches during the trial phase. By the time they formally entered the market, they had established customer networks, both stable B2B contracts with flexible B2C sales (Theme 8). This kind of customer relationship involves a mix of formal demands that must be fulfilled for business partners, alongside another customer base that also needs the products but does not necessarily plan their orders. In both categories, many of these customers were the first to support the entrepreneurs when they began selling, so their demand is seen as a priority. This dynamic reflects a form of relational engagement, where trust and interpersonal commitment drive customer relationships, as captured in Theme 5.

Their business survival and growth depend on the customers they serve, and at the same time, they also need to grow. The approach relies heavily on the trust of existing customers through the belief that they will continue the relationship and keep coming back. Because of this, the entrepreneurs do not have formal marketing strategies. They simply trust that income will come. This makes it seem as if profit is not the priority. This perspective was echoed by all informants, each in their own words, but often captured in a sentiment like: “Profit is not my first thing”, “If money is the first thing, success will not come”, “As long as you build a good relationship and love what you do, money will follow.” Thus, growth is very organic, and expansion becomes a blurry line when trying to determine exactly when market entry occurred and under what conditions.

Informants’ experiences revealed that distinct phases did exist, but in practice, they overlapped and were shaped by ongoing feedback loops, allowing learning and adaptation through each iteration. The trials were often small and conducted in informal settings, serving as stepping-stones toward more structured ventures. These experiments generated insights that guided subsequent steps as informants’ confidence grew. This reflects a feedback-driven progression in which action preceded strategy, and opportunities were discovered through ongoing engagement, responsive to evolving conditions. Importantly, the absence of deliberate sequencing does not imply a lack of purpose. Informants were engaged in meaningful action, guided more by personal relevance and contextual feedback than by formalized intent. Trying to fit such processes into rigid stage models risks overlooking the nuanced realities that characterize how SE actually develops.

Proposition 2: A dynamic loop of experiential and practice-led actions, rather than sequential stages of venture creation, can be the pathway through which SE unfolds.

This proposition contributes to SE theory in two keyways. First, it advances the previous interpretive view of SE by showing that sustainability can emerge through everyday practice without being strategically predefined ( Proposition 1). Second, it emphasizes how SE pathways are shaped by context and relational feedback, developing through ongoing interaction with lived environments rather than through sequential design. It extends processual perspectives in SE (e.g., Muñoz & Dimov, 2015) by illustrating how ventures evolve through non-linear, emotionally anchored engagement tied to lived concerns. Theoretical models that assume an ordered progression of sustainability dimensions (e.g., Belz & Binder’s Convergent Model) are insufficient to fully account for the realities observed in this context.

### Firm-level insights (Themes 3, 4, 5, 6, 7, and 8)

While much of the SE literature emphasizes the integration of sustainability principles into business models and organizational strategies (e.g., Schaltegger & Wagner, 2011; Belz & Binder, 2017), less attention has been given to how firm-level routines and structures emerge in early-stage or resource-constrained contexts. The dominant assumption is that firms integrate the TBL through deliberate structuring. However, this assumption presupposes formalization, institutional capacity, and long-term strategic vision.

In contrast, the informants in this study demonstrate that sustainable practices in their firms can evolve organically, through routinized actions, informal structuring, and context-responsive organizing principles. This echoes Muñoz & Dimov’s (2015) idea of ‘configured through doing’ approaches, suggesting that sustainability arises not through preformulated design or the implementation of a framework but through embedded practices and responsive adaptation. These experiences of the informants thus prompt a reconsideration of what it means for a firm to be ‘sustainably organized,’ shifting the focus from planned systems to evolving relational and operational logics. The organizations were not codified through business plans or legal frameworks but emerged through trust-based relationships, informal division of labor, and shared narratives of purpose. This reflects a form of organizing without organization ( Weick, 1995), where structure is relationally enacted rather than administratively designed.

The logic of effectuation ( Sarasvathy, 2001) is also apparent in how informants structured their firms by leveraging means at hand and adapting goals based on unfolding circumstances (Themes 6 and 8). Their decisions were not made to fit an ideal model but to meet immediate needs through actions. Moreover, elements of Entrepreneurial Orientation (EO)( Lumpkin & Dess, 1996) such as autonomy and proactiveness were embodied not in formal governance systems but in decentralized responsibility and flexible tasking. These insights suggest that at the firm level, SE may rely more on adaptive, rather than strategic alignment. This reinforces calls in the literature to recognize organizing as a situated and emergent process ( Welter, 2011; Muñoz & Dimov, 2015).

One of the clearest expressions of how SE took shape at the firm level was the emergence of internal routines, particularly as informants sought to stabilize operations while remaining responsive to contextual demands (Themes 3 and 5). What began for many of these ventures as improvised, solo actions eventually took on routinized and relational form. These practices also echo the logic of bricolage ( Baker & Nelson, 2005), where routines are not refined for efficiency but constructed resourcefully from available resources. From Themes 3 to 8, we can see that routine-making in SE is not merely a path to organizational stability but a reflection of values-in-practice. When conducted consistently in resource-scarce environments, such as those in the Global South, these routines help build trust and credibility with customers and partners ( Rivera-Santos et al., 2015), particularly where formal institutions are weak and relational engagement is key to business continuity ( De Castro et al., 2014; Khavul & Bruton, 2013). These firm-level routines served not only to stabilize operations but also reinforced the informal, trust-based logics through which these businesses were sustained.

In this study, the hydroponic ventures most closely resemble what Miller (1983) termed ‘simple firms’: small, informally structured, and often led directly by the founder. These ventures typically lack formal departments or systems but demonstrate flexibility and speed in decision-making, which enables them to adapt quickly to contextual shifts. This structural simplicity, often viewed as a liability in formal entrepreneurship models, appears in this context as a source of relational strength and operational responsiveness.

While the EO traits can be observed within the firm-level insights of the informants in this study, their cases offer new insight into how EO manifests in informal, resource-constrained contexts. The dominant EO literature tends to associate entrepreneurial behavior with formal strategic posture, structured decision-making, and internal alignment. In contrast, the firms in this study operated through adaptive, relational organizing. These practices reflect what Crossan et al. (1999) describe as intuitive and interpretive learning, where knowledge is shared and enacted without necessarily becoming codified into formal organizational routines. Rather than institutionalized procedures, they reflect what Miner et al. (2001) identify as organizational improvisation, a learning mode that thrives in dynamic, uncertain contexts. The routines observed here were flexible and deeply relational, oriented toward maintaining consistency and reliability in customer engagement rather than internal efficiency or scale (Theme 5).

These ventures operated on what could be termed relational reliability, a form of trust-based consistency grounded in repeated social interaction and adaptive responsiveness, echoing ideas of relational embeddedness ( Uzzi, 1997). For example, J emphasized how consistent communication through WhatsApp and follow-ups built long-term trust with buyers, even without formal service guarantees. T and D described how maintaining a stable delivery rhythm, even when harvest yields fluctuated, became key to retaining customers. Their situation signaled dependability to customers and led to informal word-of-mouth referrals, in addition to reinforcing trust with existing buyers. The relational concept captured here reflects the idea that trust and exchange are embedded in social ties rather than formal systems. These forms of consistency became substitutes for formalized contracts or customer service protocols.

Another defining insight at the firm level lies in how entrepreneurs created structural forms to support operational continuity without resorting to formal hierarchy or institutionalized procedures (Theme 7). These ventures rarely established formal departments or written protocols; instead, they relied on role fluidity and task-sharing grounded in interpersonal trust. They simply discussed tasks as they came up and adjusted based on the situation. The firm structure was less about formal designation and more about practical responsiveness, shared understanding, and flexibility. They also show that capacity-building is conducted without rigid formalization.

These patterns align with the effectuation logic ( Sarasvathy, 2001), where firms are built not on predicted futures but on adapting existing relationships and resources toward evolving goals. Structuring, then, becomes a practical means of keeping the business responsive and functional, one that privileges flexibility, responsiveness, and embedded social accountability over efficiency or scalability. The findings suggest that the structural resilience of SE can emerge from the capacity to organize fluidly in dynamic contexts. This invites reconsideration of the expectation which often implicit in stage-based and formal SE frameworks that firm-level sustainability requires increasing formalization.

Beyond routines and structures, the firm-level practice of SE was also shaped by implicit organizing principles or the underlying logic that guided how decisions were made, resources allocated, and priorities negotiated. In contrast to mission-driven enterprises that explicitly define sustainability in their core strategy, these hydroponic ventures embedded sustainability into how they operated, not what they declared. This was especially evident in Themes 4 and 8, where sustainability was not treated as a goal to be achieved but as a mode of working.

Organizing principles emerged not from frameworks, but from experience. Across these ventures, navigating trade-offs became part of the daily work of sustaining the enterprise. Tensions between product quality and cost-efficiency, between serving low-income communities and maintaining financial stability, or between short-term necessity and long-term learning were not resolved through strategy, but continuously recalibrated through decisions about pricing, partnerships, and operations (Themes 4, 6, 8). These ventures functioned as spaces of experimentation, where purpose and survival were actively negotiated.

These findings resonate with Muñoz & Dimov’s (2015) call to understand SE as an ‘ongoing organizing process,’ and challenge models that assume coherence and intention from the outset. Although the balance of the traditional TBL was temporal, negotiated, and often fragile, as it was not positioned at the strategic level of the firm, it was no less real. Those everyday decision makings were not abstract values but situated responses to real constraints and evolving relationships. For example, the informants described how they regularly adjusted planting schedules, delivery routes, or pricing strategies in response to customer feedback, resource limitations, or shifts in household responsibilities. Such adjustments emerged from ongoing negotiation toward workable outcomes.

Together, these findings extend the previous two insights by showing how personal concerns ( Proposition 1) and experiential adaptation ( Proposition 2) are sustained through firm-level mechanisms that remain informal, flexible, and deeply relational. These understanding challenges linear growth assumptions and contribute to a growing body of work (e.g., Welter, 2011; Muñoz & Dimov, 2015) that views the firm as an evolving system, where sustainability is not implemented but enacted, not planned but practiced. We underscore the importance of understanding how SE is materially organized in context-specific and adaptive ways, particularly in Global South environments marked by uncertainty and resource constraints.

Proposition 3a: SE can be sustained at the firm level through emergent routines, informal but resilient structures, and situated organizing principles that reflect adaptive, relational, and experiential logics, even in the absence of formal strategic systems or growth-oriented blueprints.

### Contextual environment-level insights (Themes 4, 6, and 8)

While SE literature continues to expand, much of it remains anchored in perspectives from developed economies ( Contreras & Dornberger, 2023). A persistent imbalance is evident in recent reviews (e.g., Abbas & Bulut, 2024; Bonfanti et al., 2024), which highlight how academic production from the Global North continues to disproportionately shape the field. The imbalance risks reinforcing assumptions that SE patterns are universally applicable.

Contextual factors are increasingly acknowledged not only in SE but also in broader entrepreneurship studies to understand entrepreneurial behavior. Foundational contributions by Welter (2011) and Zahra et al. (2014) call for deeper contextualization of entrepreneurship theory, emphasizing that entrepreneurship cannot be fully understood without considering its institutional, spatial, social, and temporal dimensions. This study takes these calls for contextualization seriously and engages with these perspectives by viewing the experiences of Jabodetabek’s hydroponic informants as inseparable from the Global South context in which they are embedded. Infrastructural unreliability, limited public support, reliance on informal social networks, and generally low public and institutional awareness of sustainability agendas are among the defining features of the Jabodetabek context, which are features that also echo other Global South situations. These conditions constrain but also enable how SE is enacted. Such constraints become sources of innovation and adaptation. As Themes 4, 6, and 8 illustrate, SE was not a product of institutional alignment but of improvisation, relational embeddedness, and adaptive problem-solving.

In parallel, the transitions literature, notably the work of Geels (2002) and Geels & Schot (2007), offers a widely cited typology of sustainability transitions, conceptualized through the interplay of niches (protected spaces for innovation), regimes (dominant socio-technical systems), and landscapes (macro-level external pressures like climate change or political shifts). While influential, their framework is largely grounded in Global North contexts, where transitions are typically supported by robust governance, institutional coordination, and well-developed infrastructure. These dynamics underscore the need for conceptual tools that can explain how sustainability transitions occur through embeddedness, relationship-based adaptation, and the contextual co-production of SE.

Three interrelated dialogues guide the interpretation of the findings in this contextual-level discussion. First, institutional voids ( Khanna & Palepu, 2010) refer to the absence or weakness of formal institutions. Second, the entrepreneurial ecosystems ( Isenberg, 2010; Stam, 2015), which in the Global South are not dense and enabling but patchy and adaptive. Third, geographic perspectives on sustainability transitions ( Geels, 2002), which often emphasize policy-led change.

Institutional voids in the Global South explain the absence or weakness of formal institutions such as regulatory systems, property rights, and contract enforcement. These voids do not merely constrain entrepreneurial action; they create opportunities for improvisation, relational solutions, and pragmatic problem-solving. In the informants’ experience, the lack of formal land tenure did not inhibit activity. For example, J cultivated on a former dumping ground based on verbal agreements and neighborly trust. By doing so, he transformed a neglected space into productive land while simultaneously preventing further public misuse, a dual contribution to urban and environmental well-being. In terms of mobility, traffic unpredictability and limited infrastructure did not disrupt delivery; instead, service was maintained through informal routines and local communication networks. These micro-adjustments, while seemingly ordinary, reflected the structural limits and adaptive ingenuity required to sustain operations in Jabodetabek’s institutional landscape.

Sustainability in the informants’ experience did not stem from alignment with ecological regulations but was embedded in everyday responses to infrastructural limits. For instance, D modified his irrigation system to reduce water dependency, T shared wells with neighbors, and J constructed shade systems using second-hand materials. These responses (Theme 4) reflected resilience through practical adjustments, not formal sustainability planning.

Institutional voids also reshaped how entrepreneurs accessed markets and maintained operations. Renting stalls to test products relied on social ties rather than formal contracts. Marketing was replaced by community-based referrals. In the absence of regulatory enforcement, practices like pesticide labeling or certification were skipped, yet accepted locally. Social capital replaced marketing infrastructure, and adaptation extended beyond technical improvisation to economic and social domains. As Zahra et al. (2014) argue, institutional heterogeneity produces divergent entrepreneurial logic. In this context, those logics are rooted in adaptive engagement, not institutional compliance.

Theories on entrepreneurial ecosystems ( Isenberg, 2010; Stam, 2015) often assume the presence of enabling institutions, networks, and capital flows. In contrast, entrepreneurial ecosystems in the Global South are patchy, adaptive, and relationally anchored ( Welter, 2011). Jabodetabek’s hydroponic entrepreneurs operated in low-density networks with limited formal mentorship or investor engagement. Their ecosystems were held together by informal referrals, trust-based exchanges, and learning-by-doing. In the limitation of the entrepreneurial ecosystem, relational trust and horizontal accountability substitute for institutional coordination. All informants built their customer bases by consistency, not promotion. They used WhatsApp and informal local communities to maintain client engagement. Trust-based arrangements replaced strategic partnerships. Theme 8 was enacted through social capital rather than financial capital or institutional brokerage.

Perspectives on sustainability transitions often emphasize policy-led changes such as systemic change driven by state actors or industrial shifts ( Geels, 2002). Yet, as Contreras & Dornberger (2023) and Van Berkum (2023) suggest, these models underrepresent how sustainability transitions emerge in informal or fragmented environments. In Jabodetabek, transitions were not policy-led but rooted in adaptive routines as a response to personal concerns. Entrepreneurs like A, J, D, and T did not articulate sustainability goals but generated sustainability outcomes such as reduced packaging, water reuse, low emissions, healthy vegetables, and low pesticide usage through day-to-day constraints and social responsiveness. Theme 6 revealed how sustainability was an unintended but emergent product of coping with scarcity, not an explicit environmental goal. In this context, transitions emerge incrementally through localized, practice-based adjustments. This reframes sustainability as co-produced through grounded routines rather than formal strategy, offering a lens to extend transition theory toward informal entrepreneurial ecosystems.

Together, these findings illustrate that SE in the Global South is not merely influenced by environmental context; it is constituted through it. Far from being anomalies, the logics offer alternative ways of organizing sustainability that differ fundamentally from formal strategic systems assumed in Global North literature.

Proposition 3b: SE is actively shaped by environmental context. In the Global South context which is marked by institutional voids, fragmented ecosystems, and urban informality, SE emerges not through formal strategic alignment, but through adaptive engagement, relational organizing, and informal structuring in response to systemic uncertainty and scarcity. These environmental dynamics function as enabling constraints that shape, rather than simply limit, the entrepreneurial pathways through which sustainability is enacted.

### Merging Propositions 3a and 3b into a Unified Proposition 3

Although propositions 3a and 3b stem from different analytical levels, they capture interconnected dynamics of the same phenomenon. The organizing logic at the firm level (3a) is both shaped by and responsive to the structural environment (3b). Thus, their mutual constitution justifies merging them into a single, coherent proposition.

Proposition 3: Being a context-responsive practice, SE is shaped by the environmental context in which it occurs.

### Conceptual Model

**Figure 1.**
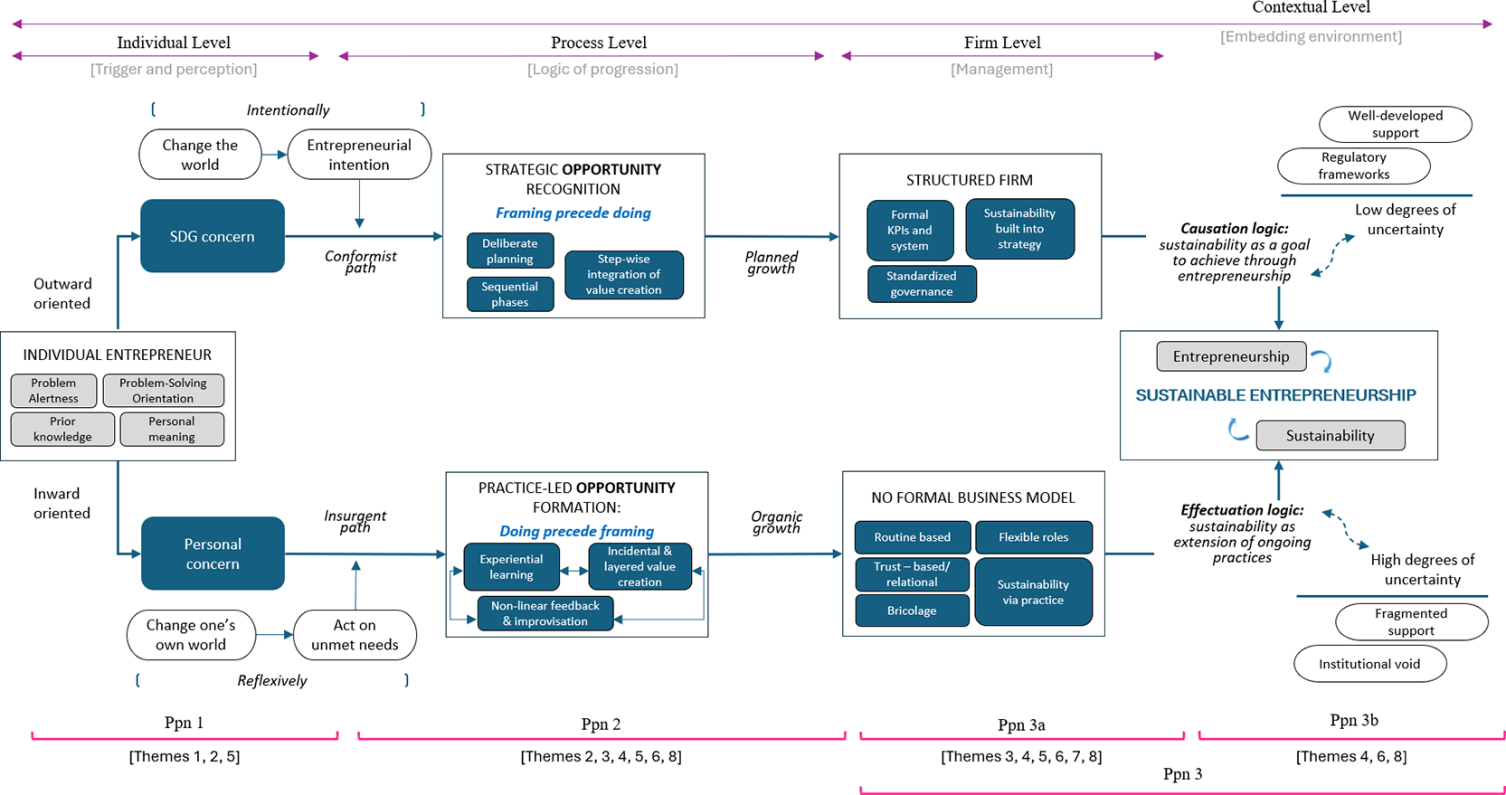
Conceptual model of SE emergence.

Figure 1 presents this study’s findings as a conceptual model of SE emergence across four interrelated levels: individual, process, firm, and contextual, arranged in a left-to-right progression. The model juxtaposes the conformist, causation-led pathway emphasized in extant literature with the insurgent, effectuation-led pathway uncovered in this study. Both pathways generate sustainability outcomes and are forms of entrepreneurship, thus constituting SE.

At the individual level, the SE pathway is shaped by orientation. An outward sustainability orientation combined with entrepreneurial intent leads to a conformist trajectory, reflecting an intention to change the world. An inward orientation, driven by personal concerns leads to an insurgent pathway, representing a reflexive effort to change one’s own world. This mechanism underpins Proposition 1.

At the process level, framing precedes doing in the conformist path. That is, entrepreneurs engage in strategic opportunity recognition, followed by solution development and business model design in a structured sequence. The growth that follows is planned. In the insurgent path, doing precedes framing. That is, opportunities are created through practice. SE action unfolds via a dynamic loop of experimentation and feedback, and the growth that follows is organic. This reflects how SE action can emerge and evolve without fixed stages. This mechanism underpins Proposition 2.

At the firm level, the planned growth path leads to structured business development, with sustainability incorporated into formal planning and strategy. Meanwhile, the organic growth path unfolds via non-structured organizing practices, embedding sustainability through emergent routines, bricolage, and relational logics, rather than through a formal business model. This mechanism underpins Proposition 3a.

At the contextual level, the pathway of SE is shaped recursively by the degree of uncertainty of the environment in which it takes place. In low-uncertainty contexts, decision-making tends to follow causation logic: sustainability is pursued as a predefined goal through deliberate entrepreneurial action. In high-uncertainty contexts (e.g., Jabodetabek), decision-making tends to follow effectuation logic: sustainability emerges as an extension of ongoing practice and adaptive problem solving. This mechanism underpins Proposition 3b, which is subsequently merged with Proposition 3a to become Proposition 3.

### Implications of this study

This study demonstrates that SE in high-uncertainty environments unfolds as a living, adaptive practice rather than a formal strategy aligned with predefined SDG agendas. This insight extends SE theory by reframing venture origins to include insurgent, practice-led pathways alongside conformist, planned approaches; challenging staged models with evidence of non-linear, emergent processes; introducing a dual-path model that encompasses both structured and adaptive trajectories; and demonstrating that constraints often treated as barriers can function as generative niches for innovation.

A key practical implication is that support for SE must begin by reflecting on the context in which they operate. In low-uncertainty settings, causation-driven approaches with strategic frameworks may be effective, whereas in high-uncertainty contexts like Jabodetabek, support programs should prioritize experimentation and reflexive learning over prescriptive business plans. Policymakers should recognize informal organizing practices as legitimate engines of SE and invest in infrastructure that enables agile experimentation.

### Limitations and recommendations for further research

While rich in situated insights, this qualitative study is limited to one UA sector and a modest number of cases. Future research could test the proposed model across regions and sectors by employing quantitative or mixed methods, as well as longitudinal or comparative research designs. Examining cultural dimensions (e.g., trust mechanisms as part of local wisdom) and tracking the evolution of insurgent SE ventures over time would deepen the understanding of how SE trajectories unfold beyond their emergence.

## Ethical Considerations

Ethical approval for this research was granted by the Research Ethics Committee of the Faculty of Economics and Business, Pelita Harapan University (Reference No. 034/DRM/EC/VII/2025), where four of the researchers are affiliated. A case study protocol was established to ensure methodological consistency and uphold ethical integrity throughout the multiple-case design, data collection, and researcher–informant interactions. All informants were briefed on the study’s objectives, the voluntary nature of their participation, data confidentiality, and handling procedures. Informed verbal consent was obtained and audio recorded before each interview. All interview recordings and related materials were securely stored in a password-protected cloud drive accessible only to the research team.

## Data Availability

The anonymized transcript data underlying this study are available on Zenodo at https://doi.org/10.5281/zenodo.17462430. All documentation files are freely accessible under the Creative Commons Attribution 4.0 International license ( Pardede & Bisowarno, 2025).

## References

[ref1] Abbas MH Bulut M : Navigating the landscape of sustainable entrepreneurship research: a systematic literature review. *Discov. Sustain.* 2024;5(1). 10.1007/s43621-024-00293-4

[ref40] Aini N Bakar A Yusiana E : Profitability and Financial Feasibility Analysis of Hydroponic Vegetable Business at Bahagia Farm. *AGRITEPA Jurnal Ilmu Dan Teknologi Pertanian.* 2024;11(1):115–128. 10.37676/agritepa.v11i1.4855

[ref2] Asao T : *Hydroponics: A standard methodology for plant biological researches.* Rijeka, Croatia: InTech; 1st ed. 2012.

[ref3] Baker T Nelson RE : Creating Something from Nothing: Resource Construction through Entrepreneurial Bricolage. *Adm. Sci. Q.* 2005;50(3):329–366. 10.2189/asqu.2005.50.3.329

[ref4] Barbosa G Gadelha F Kublik N : Comparison of Land, Water, and Energy Requirements of Lettuce Grown Using Hydroponic vs. Conventional Agricultural Methods. *Int. J. Environ. Res. Public Health.* 2015;12(6):6879–6891. 10.3390/ijerph120606879 26086708 PMC4483736

[ref5] Belz FM Binder JK : Sustainable Entrepreneurship: A Convergent Process Model. *Bus. Strateg. Environ.* 2017;26(1):1–17. 10.1002/bse.1887

[ref6] Bocken NMP Short SW Rana P : A Literature and Practice Review to Develop Sustainable Business Model Archetypes. *J. Clean. Prod.* 2014;65:42–56. 10.1016/j.jclepro.2013.11.039

[ref7] Bonfanti A De Crescenzo V Simeoni F : Convergences and divergences in sustainable entrepreneurship and social entrepreneurship research: A systematic review and research agenda. *J. Bus. Res.* 2024;170:114336–114336. 10.1016/j.jbusres.2023.114336

[ref8] Charmaz K : Constructing grounded theory: a practical guide through qualitative analysis. *Sage eBooks.* SAGE Publishing;2014.

[ref9] Cohen B Winn MI : Market imperfections, opportunity and sustainable entrepreneurship. *J. Bus. Ventur.* 2007;22(1):29–49. 10.1016/j.jbusvent.2004.12.001

[ref10] Contreras F Dornberger U : Sustainable Entrepreneurship as a Field of Knowledge: Analyzing the Global South. *Sustainability.* 2023;15(1):31. 10.3390/su15010031

[ref11] Crossan MM Lane HW White RE : An Organizational Learning Framework: From Intuition to Institution. *Acad. Manag. Rev.* 1999;24(3):522–537. 10.2307/259140

[ref12] De Castro JO Khavul S Bruton GD : Shades of Grey: How do Informal Firms Navigate Between Macro and Meso Institutional Environments? *Strateg. Entrep. J.* 2014;8(1):75–94. 10.1002/sej.1172

[ref13] De Vaus DA : *Research Design in Social Research.* Sage;2001.

[ref14] De Zeeuw H Van Veenhuizen R Dubbeling M : The role of urban agriculture in building resilient cities in developing countries. *J. Agric. Sci.* 2011;149(S1):153–163. 10.1017/s0021859610001279

[ref15] Dean TJ McMullen JS : Toward a Theory of Sustainable entrepreneurship: Reducing Environmental Degradation through Entrepreneurial Action. *J. Bus. Ventur.* 2007;22(1):50–76. 10.1016/j.jbusvent.2005.09.003

[ref16] Dubbeling M Van Veenhuizen R De Zeeuw H : *Cities, poverty and food: multi-stakeholder planning in urban Agriculture.* Practical Action Pub;2010.

[ref17] Food and Agriculture Organization: *Profitability and sustainability of urban and peri-urban agriculture.* FAO;2007. Agricultural Management, Marketing and Finance Occasional Paper No. 19.

[ref18] Garud R Karnøe P : Bricolage versus breakthrough: distributed and embedded agency in technology entrepreneurship. *Res. Policy.* 2003;32(2):277–300. 10.1016/s0048-7333(02)00100-2

[ref19] Geels FW : Technological Transitions as Evolutionary Reconfiguration processes: a multi-level Perspective and a case-study. *Res. Policy.* 2002;31(8-9):1257–1274. 10.1016/S0048-7333(02)00062-8

[ref20] Geels FW Schot J : Typology of sociotechnical transition pathways. *Res. Policy.* 2007;36(3):399–417. 10.1016/j.respol.2007.01.003

[ref21] Giacomini D Muzzi C Albertini S : Micro-context and institutional entrepreneurship: Multiple case studies of innovative start-ups. *Fast Growing Firms in a Slow Growth Economy.* Edward Elgar Publishing;2016.

[ref22] Hockerts K Wüstenhagen R : Greening Goliaths versus emerging Davids — Theorizing about the role of incumbents and new entrants in sustainable entrepreneurship. *J. Bus. Ventur.* 2010;25(5):481–492. 10.1016/j.jbusvent.2009.07.005

[ref23] Isenberg DJ : How to start an entrepreneurial revolution. *Harv. Bus. Rev.* 2010;88(6):40–50.

[ref24] Khan MM Younis A Akram MT : Feeding the cities: Urban agriculture for food security and sustainability of urban areas. *CABI Reviews.* 2024;19(1). 10.1079/cabireviews.2024.0053

[ref25] Khanna T Palepu KG : *Winning in Emerging Markets: a Road Map for Strategy and Execution.* Harvard Business Press;2010;13–26.

[ref26] Khavul S Bruton GD : Harnessing Innovation for Change: Sustainability and Poverty in Developing Countries. *J. Manag. Stud.* 2013;50(2):285–306. 10.1111/j.1467-6486.2012.01067.x

[ref27] Kuckertz A Wagner M : The influence of sustainability orientation on entrepreneurial intentions—Investigating the role of business experience. *J. Bus. Ventur.* 2010;25(5):524–539. 10.1016/j.jbusvent.2009.09.001

[ref28] Lal R : Home gardening and urban agriculture for advancing food and nutritional security in response to the COVID-19 pandemic. *Food Secur.* 2020;12(4):871–876. 10.1007/s12571-020-01058-3 32837634 PMC7311182

[ref29] Linnanen L : An Insider’s Experiences with Environmental Entrepreneurship. *Greener Manag. Int.* 2002;2002(38):71–80. 10.9774/gleaf.3062.2002.su.00008

[ref73] Lumpkin GT Dess GG : Clarifying the entrepreneurial orientation construct and linking it to performance. *Acad. Manag. Rev.* 1996;21(1):135–172. 10.5465/amr.1996.9602161568

[ref30] Maaßen C Rovira R Urbano D : A Process Model for Sustainable Entrepreneurship: Evidence from a Highly Entrepreneurial European Region. *J. Soc. Entrep.* 2023;1–32:1–32. 10.1080/19420676.2023.2221262

[ref74] Magwaza ST Magwaza LS Odindo AO de : Hydroponic technology as decentralised system for domestic wastewater treatment and vegetable production in urban agriculture: A review. *Sci. Total Environ.* 2020;698:134154. 10.1016/j.scitotenv.2019.134154 31505342

[ref31] Mair J Marti I : Entrepreneurship in and around institutional voids: A case study from Bangladesh. *J. Bus. Ventur.* 2009;24(5):419–435. 10.1016/j.jbusvent.2008.04.006

[ref32] Martinez R Masron IN : Jakarta: A city of cities. *Cities.* 2020;106(102868):102868. 10.1016/j.cities.2020.102868 32863521 PMC7442427

[ref33] Matzembacher DE Raudsaar M Barcellos MD de : Business Models’ Innovations to Overcome Hybridity-Related Tensions in Sustainable Entrepreneurship. *Sustainability.* 2020;12(11):4503. 10.3390/su12114503

[ref34] Merriam SB Grenier RS : *Qualitative research in practice: Examples for discussion and analysis.* Jossey-Bass; 2nd ed. 2019.

[ref35] Miller D : The Correlates of Entrepreneurship in Three Types of Firms. *Manag. Sci.* 1983;29(7):770–791. 10.1287/mnsc.29.7.770

[ref36] Miner AS Bassoff P Moorman C : Organizational Improvisation and Learning: A Field Study. *Adm. Sci. Q.* 2001;46(2):304–337. 10.2307/2667089

[ref37] Muñoz P Cohen B : Sustainable Entrepreneurship Research: Taking Stock and looking ahead. *Bus. Strateg. Environ.* 2017;27(3):300–322. 10.1002/bse.2000

[ref38] Muñoz P Dimov D : The call of the whole in understanding the development of sustainable ventures. *J. Bus. Ventur.* 2015;30(4):632–654. 10.1016/j.jbusvent.2014.07.012

[ref39] Naresh R Jadav SK Singh M : Role of Hydroponics in Improving Water-Use Efficiency and Food Security. *International Journal of Enviornment and Climate Change.* 2024;14(2):608–633. 10.9734/ijecc/2024/v14i23976

[ref41] Orsini F Kahane R Nono-Womdim R : Urban agriculture in the developing world: a review. *Agron. Sustain. Dev.* 2013;33(4):695–720. 10.1007/s13593-013-0143-z

[ref42] Pardede G : A SWOT Analysis of the Hydroponics Entrepreneurship as Sustainable Income in Covid-19 Pandemic Adaptation. *Jurnal Teknotan.* 2022;16(2):93. 10.24198/jt.vol16n2.5

[ref43] Pardede G Bisowarno SD : Anonymized transcript data underlying “Sustainable entrepreneurship emergence as practice: A multi-level pathway model”.[Data set]. *Zenodo.* 2025. 10.5281/zenodo.17462430 PMC12914167 41716355

[ref72] Patzelt H Shepherd DA : Recognizing opportunities for sustainable development. *Entrep. Theory Pract.* 2011;35(4):631–652. 10.1111/j.1540-6520.2010.00386.x

[ref44] Pomoni DI Koukou MK Vrachopoulos MG : A Review of Hydroponics and Conventional Agriculture Based on Energy and Water Consumption, Environmental Impact, and Land Use. *Energies.* 2023;16(4):1690. 10.3390/en16041690

[ref45] Purvis B Mao Y Robinson D : Three Pillars of sustainability: in Search of Conceptual Origins. *Sustain. Sci.* 2019;14(3):681–695. springer. 10.1007/s11625-018-0627-5

[ref46] Radhy M Yusna Y Fyka SA : Business Feasibility Analysis with Hydroponic System in Kendari City (Case Study of Family Garden Hydroponic Vegetable Business). *JIA (Jurnal Ilmiah Agribisnis): Jurnal Agribisnis Dan Ilmu Sosial Ekonomi Pertanian.* 2024;9(2):186–191. 10.37149/jia.v9i2.1158

[ref47] Read S Dew N Sarasvathy SD : Marketing under Uncertainty: The Logic of an Effectual Approach. *J. Mark.* 2009;73(3):1–18. 10.1509/jmkg.73.3.001

[ref48] Resh HM : *Hydroponic food production: a definitive guidebook for the advanced home gardener and the commercial hydroponic grower.* Crc Press/Taylor & Francis Group, Cop.;2012.

[ref49] Rivera-Santos M Holt D Littlewood D : Social Entrepreneurship in Sub-Saharan Africa. *Acad. Manag. Perspect.* 2015;29(1):72–91. 10.5465/amp.2013.0128

[ref50] Rosário AT Raimundos RJ Cruz SP : Sustainable Entrepreneurship: A Literature Review. *Sustainability.* 2022;14(9):5556. 10.3390/su14095556

[ref51] Sarasvathy SD : Causation and effectuation: toward a Theoretical Shift from Economic Inevitability to Entrepreneurial Contingency. *Acad. Manag. Rev.* 2001;26(2):243–263. 10.2307/259121

[ref52] Schaltegger S Wagner M : Sustainable entrepreneurship and sustainability innovation: categories and interactions. *Bus. Strateg. Environ.* 2011;20(4):222–237. 10.1002/BSE.682

[ref53] Schwandt TA : Constructivist, interpretivist approaches to human inquiry. Denzin NK Lincoln YS , editors. *Handbook of qualitative research.* Thousand Oaks, CA: Sage Publications;1994; pp.118–137.

[ref54] Segal G Borgia D Schoenfeld J : The motivation to become an entrepreneur. *Int. J. Entrep. Behav. Res.* 2005;11(1):42–57. 10.1108/13552550510580834

[ref55] Setiyawan H Handoyo Mulyo J Irham I : Financial feasibility of NFT system hydroponic urban farming businesses in Semarang city. *BIO Web of Conferences.* 2025;158:02002. 10.1051/bioconf/202515802002

[ref56] Shane S : Prior Knowledge and the Discovery of Entrepreneurial Opportunities. *Organ. Sci.* 2000;11(4):448–469. 10.1287/orsc.11.4.448.14602

[ref57] Shane S Venkataraman S : The Promise of Entrepreneurship as a Field of Research. *Acad. Manag. Rev.* 2000;25(1):217–226. 10.5465/amr.2000.2791611

[ref58] Shepherd DA Patzelt H : The New Field of Sustainable Entrepreneurship: Studying Entrepreneurial Action Linking “What Is to Be Sustained” With “What Is to Be Developed.”. *Entrep. Theory Pract.* 2011;35(1):137–163. 10.1111/j.1540-6520.2010.00426.x

[ref59] Spence M Ben Boubaker Gherib J Ondoua Biwolé V : Sustainable Entrepreneurship: Is Entrepreneurial will Enough? A North–South Comparison. *J. Bus. Ethics.* 2011;99(3):335–367. 10.1007/s10551-010-0656-1

[ref60] Stam E : Entrepreneurial Ecosystems and Regional Policy: A Sympathetic Critique. *Eur. Plan. Stud.* 2015;23(9):1759–1769. 10.1080/09654313.2015.1061484

[ref61] Terán-Yépez E Marín-Carrillo GM del Casado-Belmonte M : Sustainable entrepreneurship: Review of its evolution and new trends. *J. Clean. Prod.* 2020;252:119742. 10.1016/j.jclepro.2019.119742

[ref62] Uzzi B : Social Structure and Competition in Interfirm Networks: The Paradox of Embeddedness. *Adm. Sci. Q.* 1997;42(1):35–67. 10.2307/2393808

[ref63] Van Berkum S : How Urban Growth in the Global South Affects Agricultural Dynamics and Food Systems Outcomes in Rural Areas: A Review and Research Agenda. *Sustainability.* 2023;15(3):2591. 10.3390/su15032591

[ref64] Van de Ven AH : Suggestions for studying strategy process: A research note. *Strateg. Manag. J.* 1992;13(S1):169–188. 10.1002/smj.4250131013

[ref65] Wahyuni S : *Qualitative Research Method: Theory and Practice.* Penerbit Salemba;2024.

[ref66] Weick KE : *Sensemaking in organizations.* Sage Publications;1995; vol.3.

[ref67] Welter F : Contextualizing entrepreneurship—conceptual challenges and ways forward. *Entrep. Theory Pract.* 2011;35(1):165–184. 10.1111/j.1540-6520.2010.00427.x

[ref68] World Bank: *World Bank Group support for small to medium enterprises (SMEs).* World Bank;2019a.

[ref69] World Bank: *Small to medium enterprises (SMEs) finance.* World Bank;2019b.

[ref70] Yin RK : *Case Study Research and Applications: Design and Methods.* SAGE Publications; 6th ed. 2018.

[ref71] Zahra SA Wright M Abdelgawad SG : Contextualization and the advancement of entrepreneurship research. *International Small Business Journal: Researching Entrepreneurship.* 2014;32(5):479–500. 10.1177/0266242613519807

