# Supplementary material for: Sustainable Entrepreneurship Emergence as Practice: A Multi-Level Pathway Model
Source: F1000Res. 2025 Nov 17;14:1263. [Version 1] doi: 10.12688/f1000research.171837.1 (PMC12914167; doi:10.12688/f1000research.171837.1)
Supplement: Peer review history

# Reviewer report: Reviewer response for version 1

**DOI:** 10.5256/f1000research.189495.r453970

**Published:** 2026-02-17

**Authors:** Ratna Lindawati Lubis

**Affiliations:** 1 Telkom University, Jawa Barat, Indonesia

Overall Assessment

 The study makes a significant contribution by providing empirical insights from an underrepresented Global South context, thereby addressing a notable gap in the sustainable entrepreneurship literature, which remains heavily skewed toward Global North settings. In particular, the identification of non–intention-led and reflexive pathways to sustainable entrepreneurship offers a meaningful conceptual extension to existing models that predominantly assume predefined sustainability intentions.

 Additionally, the use of a multi-level, practice-based analytical framework (individual, process, firm, and contextual environment) is of major significance, as it advances process-oriented understanding of how sustainable entrepreneurship emerges in real-world conditions characterized by institutional uncertainty.

 However, the significance of the findings is contextual rather than universal, given the limited number of cases and sector-specific focus. As such, while the article does not constitute a paradigm shift, it makes a substantial and valuable contribution to theory refinement and contextualization within the field.

Is the work clearly and accurately presented and does it cite the current literature?

Yes

If applicable, is the statistical analysis and its interpretation appropriate?

Yes

Are all the source data underlying the results available to ensure full reproducibility?

Yes

Is the study design appropriate and is the work technically sound?

Yes

Are the conclusions drawn adequately supported by the results?

Yes

Are sufficient details of methods and analysis provided to allow replication by others?

Yes

Reviewer Expertise:

Entrepreneurship Education (EE) and Education for Sustainable Development (ESD)

I confirm that I have read this submission and believe that I have an appropriate level of expertise to confirm that it is of an acceptable scientific standard.

# Reviewer report: Reviewer response for version 1

**DOI:** 10.5256/f1000research.189495.r447145

**Published:** 2026-01-13

**Authors:** Eka Sudarmaji

**Affiliations:** 1 University of Pancasila, South Jakarta, Indonesia

Comment 1: The manuscript provides a wealth of information regarding the four hydroponic farmers, as presented in Tables 2, 3, 4, and 6. While this demonstrates a comprehensive data-gathering process, the connection between the specific details of lettuce production and monthly revenues and the overarching concept of Sustainable Entrepreneurship may not be immediately apparent to the reader. The relationship between the individual farming narratives and the theoretical contributions could be more explicitly defined.

 Comment 2: The manuscript employs several specialized terms, including "effectuation logic," "institutional voids," and "bricolage." While these are pertinent to the subject matter, brief definitions would enhance accessibility for F1000Research's diverse readership.

 Comment 3: Regarding the flow of the Discussion, it covers a lot of territory: eight themes, four levels of analysis, three propositions, and two pathways. While this complexity highlights the depth of the findings, the presentation sometimes obscures the central argument. The analysis is compelling, yet the delivery could be more direct.

Is the work clearly and accurately presented and does it cite the current literature?

Yes

If applicable, is the statistical analysis and its interpretation appropriate?

Not applicable

Are all the source data underlying the results available to ensure full reproducibility?

Yes

Is the study design appropriate and is the work technically sound?

Yes

Are the conclusions drawn adequately supported by the results?

Yes

Are sufficient details of methods and analysis provided to allow replication by others?

Yes

Reviewer Expertise:

I look at this text with a critical eye because I am a data expert who specializes in the energy economy. I saw major methodological flaws right away: using four hydroponic cases to say that there are universal patterns of sustainable business raises real questions about external validity. Because I've studied econometrics, I can spot problems that qualitative analysts often miss, such as selection bias, omitted variables, and issues with determining cause and effect. Because I know a lot about how people adopt new technologies and behavioral economics, I can ask whether "personal concern" drives sustainability or just goes along with it. I check not only the consistency of the story but also the reliability of the data. This makes me the only person who can say for sure whether these qualitative results can withstand scientific analysis.

I confirm that I have read this submission and believe that I have an appropriate level of expertise to confirm that it is of an acceptable scientific standard.
